# Permitting patients to pay for participation in clinical trials: the advent of the P4 trial

**DOI:** 10.1007/s11019-016-9741-2

**Published:** 2016-10-18

**Authors:** David Shaw, Guido de Wert, Wybo Dondorp, David Townend, Gerard Bos, Michel van Gelder

**Affiliations:** 1 Department of Health, Ethics and Society, CAPHRI Research School, Maastricht University, Maastricht, The Netherlands; 2 Maastricht University Medical Centre, Maastricht, The Netherlands; 3 Institute for Biomedical Ethics, University of Basel, Basel, Switzerland

**Keywords:** Clinical trials, Research ethics, Participatory research, Exploitation, Patient participation, Crowdfunding

## Abstract

In this article we explore the ethical issues raised by permitting patients to pay for participation (P4) in clinical trials, and discuss whether there are any categorical objections to this practice. We address key considerations concerning payment for participation in trials, including patient autonomy, risk/benefit and justice, taking account of two previous critiques of the ethics of P4. We conclude that such trials could be ethical under certain strict conditions, but only if other potential sources of funding have first been explored or are unavailable.

## Introduction

Imagine that you are suffering from terminal cancer and you’ve heard about a clinical trial that aims to test a promising new drug in humans for the first time. You’re keen to enroll as your current treatment is no longer effective. On reading the information sheet and consent form, you are surprised to see that participation in the trial will cost 20,000 Euros because of the high cost of the drug involved. Is it ethical to ask and permit patients to pay for participation (P4) in clinical trials? [P4 medicine is defined as predictive, preventive, personalized, and participatory (Hood and Flores 2012); P4 trials could be an important part of this emerging trend.] Sometimes this is the only way to secure funding for clinical research, but asking patients to do this raises important ethical issues. In this article, we identify and analyze these issues, assess the ethical acceptability of asking patients to pay for participation in trials, and discuss whether there are any categorical objections to this practice, before making several recommendations regarding the conduct of P4 trials. We also respond to two previous critiques of P4 by Emanuel et al. (2015) and Wenner et al. (2015). Our discussion is relevant to patient payment for any type of clinical trial, but our focus is on cancer research. Certain specifics in the analysis regarding payment for participation will vary according to the disease in question, but the core issues remain the same.

## Autonomy and justice: categorical objections

When faced with the proposal to ask patients to pay for participation in clinical trials, some people might reflexively react that this is simply unethical, even without detailed exploration of the relevant issues. There are two main reasons that might underpin this stance. First, patients in general are a potentially vulnerable group, and involving them in research is already ethically challenging even under normal circumstances. Terminally ill patients are a particularly vulnerable group, and asking such patients to contribute considerable sums in exchange for involvement in research raises serious concerns about autonomy and exploitation. Under what conditions should an individual be judged competent to invest large sums in an uncertain research enterprise? Furthermore, while some patients are well-off, others are very poor, and might incur large debts by borrowing substantial sums in order to participate in a trial (assuming that they had sufficient collateral to secure a loan). Emanuel et al. (2015) argue that what they call “pay to play” trials should be prohibited, in part because of such considerations:

Pay-to-play research is less likely to be a collaborative partnership than a psychological exploitation of individuals desperate to do anything to save their own or a loved one’s life. Although willingness to pay might indicate understanding and voluntariness by participants, it might also reveal unrealistic expectations and undue pressure. The chances for success of early phase experimental drugs are much smaller than either researchers or laypeople think. The vast majority of experimental agents, ~90 %, that enter human trials even under current circumstances fail primarily for reasons of safety and efficacy.

These are serious concerns, but they are not of a categorical nature: while it is true that asking for payment may contribute to the therapeutic misconception, terminally ill patients are usually judged competent to give consent to enter a potentially risky clinical trial, and it is not obvious that adding a fee to the equation changes things fundamentally. Furthermore, even if it were shown that P4 trial participants were more prone to the therapeutic misconception (where participation in research is mistaken for involvement in clinical therapy), steps other than prohibition could be taken to address this. For example, patients could be informed that 90 % of new drug trials fail, and reminded very clearly that seeking clinical benefit from such a trial is likely to be a futile endeavour (see “Risk, benefit and trial design” section). Taking these extra steps would address the worry of Emanuel et al. that participants are “likely to overestimate the chances of a successful outcome.” Ironically, Emanuel et al. themselves fall victim to the therapeutic misconception when considering the social value of P4 trials; they state that “buying into a clinical trial ultimately constitutes the purchasing of a good or service that happens to contribute social value as a side effect.” But this gets things the wrong way round: those who “pay to play” are paying for participation in a trial that aims to provide social value through generalisable knowledge. The goods and services they pay for are involvement in research, with all its uncertainties; any benefit to the patient/participant is actually a side effect.

Emanuel et al. also raise concerns about voluntariness:

Desperate patients are likely to feel pressured to pay for participation, leading to biased decisionmaking that raises questions about voluntariness…Just because people can pay—or raise— substantial sums does not mean they cannot be taken unfair advantage of, especially if their illness or that of a loved one compromises or biases their decisionmaking. Typically, in such circumstances, society protects individuals from potential abuse.

Once again, Emanuel and colleagues assume, from a presumption of likely exploitation, that prohibition is the appropriate response to these concerns—despite the fact that illness can bias decision-making in any patient considering participation in a phase 1 trial. The fact that people are paying for participation does not mean that they *are* being taken advantage of, even if the risk of exploitation is increased. As Emanuel et al. themselves state, society takes steps to protect individuals from potential abuse in clinical trials, and these steps might well be sufficient to prevent any added risk of exploitation of or bias in potential participants in P4 trials; if they are not sufficient to this new challenge, these measures could be extended or enhanced. It is true that asking patients for payment adds another factor that could increase pressure on patients, but it is not obvious that prohibition is the appropriate response to this added factor.

The second objection is that it is simply unjust to ask anyone to pay for participation in clinical research (even if they become stakeholders in the research enterprise by doing so). However, this too is not obviously true, and even if it were, this might again not be sufficient reason to prohibit the practice categorically. If patients want to take part in a research project and the project simply will not be funded without their participation, it might well be more unjust to deny them the opportunity to contribute, even if this raises further issues of justice that we will now turn to. (It might be argued that it is not unjust to deny patients the opportunity to pay for participation in research that has failed to secure funding in the normal way because this failure is suggestive of lower-quality research. However, many excellent trials cannot obtain funding, and failure of market mechanisms alone is not an indicator of a poor research proposal.)

If it is ethical in terms of individual autonomy and risk to ask patients to pay for participation in a trial, is it fair to do so when only well-off patients will be able to afford the expense? In essence, only the well-off have a chance of benefitting directly from such a trial (Emanuel et al. 2015). Some would argue that it would be contrary to principles of equity to permit such a trial, as it entails complicity of the research process with preserving certain social advantages that are not enjoyed by socioeconomically disadvantaged people; indeed, “fair participant selection” is one of eight suggested principles for ethical clinical review (Emanuel et al. 2008). The FDA mentions justice concerns in its overview of the 2009 rule concerning payment for participation, though not under the heading of ethical considerations:

A significant concern (…) relates to the potential effect on access to investigational therapies for economically disadvantaged individuals and the uninsured. Allowing sponsors to charge could impose a significant financial burden on many seriously ill individuals who lack therapeutic alternatives and could preclude access by some needy patients. However, in the past, many companies that have provided investigational drugs for treatment use have often included assistance programs to cover the costs for those who could not otherwise afford them. FDA expects this practice will continue. (FDA 2009)

However, the focus of this passage appears to be on expanded access outside trials rather than charging research participants: while pharmaceutical companies do indeed sometimes provide financial support for compassionate use programmes, such support has not generally been available in a trial of a very expensive drug where patients are being asked to pay for participation.

One response to these justice objections would be to argue that it is fair enough to let well-off people use their money to participate in a trial, as they are very unlikely to experience any therapeutic benefit, meaning that poorer people are not actually any worse off for non-participation (and indeed may be better off as they don’t need to assume any of the risk). Indeed, the correct terminology is really payment for participation in research, not payment for clinical care or for access to a therapeutic dose of a drug. Anyone who thinks in the latter terms is a victim of the therapeutic misconception.

Another response is that future worse-off people who suffer from the same disease will may well eventually benefit if these rich people contribute towards paying for a trial that will not take place unless funds are raised in this way. This Rawlsian argument is quite persuasive; no-one will benefit if the trial never goes ahead, but if it is financed by well-off participants, socioeconomically disadvantaged patients are quite likely to benefit from any resulting new drug at some point in the future (though it will probably not be the same disadvantaged patients who end up benefiting, but different ones in the future). Would it not be counterproductive to prohibit this type of research funding mechanism simply because it represents a short-term disadvantage for the worse-off? If the worst-off in society tend to benefit in the long run, a temporary inequality to those who cannot afford to participate may be justified [see Rawls (1999) for more background on the difference principle].

This argument may invite the practical criticism that, if the drug in question is so expensive, it is unlikely to be approved for funding by national health systems and insurers, meaning that poor people will not in fact be able to access it. If correct, this would mean that the reason for asking people to pay to participate in research—the cost of the drug—could itself mean that the research is rendered largely irrelevant, as the investigative agent will remain too expensive to fund even if licensed and mass-produced. Generally, the potential to provide “social value” is a requirement for any research, and a drug that will not be covered by a national health system or an insurer is unlikely to meet this criterion. However, having participants fund trials might actually lead to cheaper outcomes by circumventing industry: there would be no need for expensive patents if patients pay for part of the development process of drugs. Indeed, another incentive to have participants pay to join a trial could be a share in the profits from any resulting drug. Overall, it seems quite likely that the financial contribution made by well-off participants in research could yield substantial long-term benefits for society, including for the worse-off. If the Rawlsian justification for allowing patients to pay for participation is regarded as important, an additional criterion for such studies could be that the drug must at least have a chance of being cheap enough to be widely funded if it reached the (less expensive) mass-production stage.

But what if this justice-preserving condition cannot be met? Some might argue that this Rawlsian justification is not really required in any case. Why is it unacceptable for the rich to profit from research that they themselves pay for without others also profiting in the long run? Can’t they do as they please with their own money? Given the principles of contemporary economics (and indeed of capitalism), it might seem strange to protest against this “research for and by the rich”. The problem with this argument is that a great deal of public money is invested in the medical research infrastructures of most countries. While public/private partnerships are increasingly common, hospitals and doctors tend to be paid for by the state, or at least by mutual insurance schemes; also, much of the upstream science upon which the downstream advances are based flows from publicly-funded research. Allowing rich people preferential access to clinical trials because of the ability to pay would be unfair as long as public resources are also involved in the research. In this argument, only if rich potential participants paid for the “whole show” in clinical trials, including everything from hospital costs to administration fees to nurses’ and doctors’ salaries, could their paying for the investigative agent be acceptable.

Another problem occurs at the other end of the financial scale: is there not a risk that poor people will get themselves into serious debt in order to enroll in a paying trial? As mentioned above, even rich patients could be in a vulnerable state and might not be fit to make such a serious financial decision; for patients living in poverty, loans might be the only way to raise funds to participate (and some might not even be able to secure loans). Theoretically, safeguards could be designed to prevent such situations arising, but preventing the worst-off from enrolling in a trial because they had to borrow money to do so raise further justice concerns.

Finally, it is also worth considering how the identity of the sponsor could affect the ethics of a P4 trial. While it might seem problematic for a pharmaceutical company to seek financial assistance from participants in developing a drug that it may ultimately profit from, there would presumably be less concern about a patient advocacy group acting as a sponsor as its goal would be purely to help patients.

## Risk, benefit and trial design

In addition to concerns regarding autonomy and justice, subtly different issues of risk and benefit are in play depending on the type of trial in question. The first issue is that phase 1 (first-in-human) trials are designed to assess safety not efficacy. Patients in phase 1 trials are very likely to receive a sub-therapeutic dose due to the gradual escalation from very low doses throughout the trial (Le Tourneau et al. 2009). Most people who paid to take part in such a trial could not receive any benefit from their involvement, and (as mentioned above) by asking them to pay, researchers could further reinforce the therapeutic misconception, putting patients at risk of exploitation. Also, if the drug in question had low anticipated toxicity and could easily be tested in healthy volunteers, asking terminally ill patients to pay to participate in that phase would be an even more obvious case of exploitation.

Even in modern dose escalation trial designs some would still receive subclinical amounts of the study drug. However, it might be regarded as acceptable to let people pay to participate in a trial if there were *a minimum chance of receiving a potentially therapeutic dose*; perhaps 75 % or higher. This could represent an acceptable compromise between “buying benefit” in a trial, which is unrealistic, and the meager “purchasing participation” in research, which is unlikely to be attractive to those who seek to access the claimed potential benefits of the drug. Even in such a trial, though, some patients would pay high amounts to be used as test subjects with no prospect of benefit. Moreover, increasing the number of patients exposed to higher doses also increases the risk of toxicity for each of them. (In addition, patients would very possibly be giving up palliative or other drugs of known efficacy to join such a trial; while this also true of normal phase 1 trials, in this case they would be paying for the privilege of abandoning proven treatments.) However, if sufficient safeguards against the therapeutic misconception were put in place and the risks explained adequately, there appear to be no conclusive arguments against allowing patients to pay to participate in a phase 1 trial.

In phase 2/3 trials, all doses would be potentially therapeutic, but another problem occurs unless the study in question is a single-arm trial: in randomized controlled trials (RCTs), each patient faces a 50 % chance of being allocated to the control group. Even if patients in this group receive the current gold standard drug rather than a placebo, they will not receive the investigative agent they may mistakenly believe they are paying to access; what they are actually paying for is participation in the trial. Patients are actually less likely to receive the investigative agent in a phase 2 trial than in a phase 1 trial, even if they are more likely to receive a potentially therapeutic dose of it. In a typical phase 1 trial they will all receive the agent, but most of them at a subtherapeutic dose; in a phase 2/3 RCT, 50 % will not receive the new drug, but all of those who do receive it will receive a potentially therapeutic dose. One possible solution to this problem would be to charge only those who are randomized to receive the investigative agent, but this would require unblinding the trial, and this could compromise the study Of course, the issue of receiving the control rather than the new drug does not arise in single-arm phase 2 or 3 studies, where patients are certain to receive the investigative agent. However, researchers must be careful avoid any temptation to choose single-arm over RCT trials in order to increase the attractiveness of the trial for paying participants in cases where doing so would be methodologically unsound (Wenner et al. 2015). Two particular issues arise when we consider patients paying for participation in single-arm phase 2 trials. First, a study where everyone pays to receive a drug could be perceived as less fair than a randomised phase 2 trial that is funded by payers but also includes those who don’t pay, with a 50–50 chance of any one receiving the active agent. The latter trial would be fairer in terms of opportunity costs for the less well-off, but less fair overall in terms of gaining access to the drug. Second, those who can afford to pay for the drug will presumably come from a better socioeconomic background, meaning they are more likely to have led healthier lives; this could potentially skew outcomes in any trial, but particularly in a single-arm trial where they are the only participants.

What if trial designs were adapted to address some of these concerns? In addition to adopting an interparticipant dose escalation model (which is good practice anyway) it could be argued that, in certain circumstances like these, patients willing to pay could also give consent to a phase 1 trial where all doses are potentially therapeutic. However, this raises certain problems. Such a trial would obviously be more dangerous and be contrary to the principle of proportionality as all patients would be exposed to a higher risk of toxicity and side effects (as well as paying for this added risk). Dose escalation trials might alleviate this problem to some extent because they allow some degree of assessment of toxicity and side effects at different dose levels; using the same doses for everyone would weaken the evidence generated by the trial. Therefore, those who move to the therapeutic dose are monitored on the way to that dose and observed whilst taking it. Similarly, an even more radical suggestion would be to abandon both randomization and the direct control group in a phase 2 or 3 trial, relying on non-contemporaneous control data (i.e., the information already gathered about the gold standard treatment). This would allow everyone paying for access to the drug to receive it. But again, doing so would weaken the design of the trial substantially, and perhaps catastrophically, given the conventional paradigm that randomised double blind trials are the gold standard. Weakening the trial in this way would very probably mean that the second FDA criterion mandating that drugs used in these trials are both safe and effective was not fulfilled; if the data obtained in the trial has to be sufficient to establish that a drug is safe or effective, then the design must be good enough to ensure that the results are reliable. Abandoning accepted best practice in trial design is likely to offer less convincing evidence. While some trials would not be able to proceed without using the P4 model and modifying their design, it might be better not to go ahead if the trial cannot provide good evidence.

There would also be concerns about recruitment, discontinuation and withdrawal in any trial where patients paid to participate. Even if a trial was approved by an ethics committee, would it be able to recruit enough patients? It seems likely that patients would be less inclined to join such a trial than in the case of a typical trial where participation is free of charge. This might lead to researchers being tempted to loosen inclusion criteria to boost recruitment (although this is true of any trial). On the other hand, there are many well-off people with cancer—what if more people want to pay to join the trial than is scientifically necessary? It is normally regarded as unethical to recruit more people than required for a clinical trial, particularly a phase 1 study as it exposes more people to potential harm than is necessary; doing so also compromises the scientific integrity of the study. Again, variants of these issues occur in typical clinical trials, but paying patients may be able to apply more pressure on researchers than would otherwise be the case. Researchers might well be more vulnerable to coercion from patients in a P4 trial because patients feel they have more right to control what happens in the trial.

Other issues are raised at the other end of trial involvement. What would happen to patients who paid and joined the trial, only for the trial to be abandoned because of low recruitment? Would they be able to keep receiving the drug outside the trial? Normally they would not, but if they pay for it they might expect to. Furthermore, would patients who died during the trial or withdrew from it for other reasons have their payment refunded? Given that the drugs would have to be manufactured before the trial began, we can assume that there would be no refunds. These concerns could probably be addressed with a well-designed information sheet and consent form, but they do deserve careful consideration.

## Regulation of P4 trials

Few guidelines on P4 trials have been published. In 2009 the FDA introduced new rules permitting payment for access to experimental drugs, both within the context of clinical trials and as “expanded access” or compassionate use (when patients are prescribed drugs that are not yet licensed (FDA 2009). The rationale for permitting charging patients in the context of a trial was that some potentially beneficial research will simply never be conducted without funding from this source, due to the great expense of manufacturing the experimental drug: “cost recovery is justified in clinical trials only when necessary to further the study and development of promising drugs that might otherwise be lost to the medical armamentarium.” There was very little reaction from the medical or bioethical community to these new guidelines, despite the fact that these new regulations permit charging patients for participation in clinical trials, a practice most infamously associated with the exploitative and controversial practices of the Burzynski Clinic (Szabo 2013). This may be because the new guideline simply formalized existing FDA practice (Rossen 2009), but the silence is surprising given the ethical issues raised by this practice. The FDA sets four criteria that must be met in order for patients to be charged for participation in a trial:

(i) Provide evidence that the drug has a potential clinical benefit that, if demonstrated in the clinical investigations, would provide a significant advantage over available products in the diagnosis, treatment, mitigation, or prevention of a disease or condition;

(ii) Demonstrate that the data to be obtained from the clinical trial would be essential to establishing that the drug is effective or safe for the purpose of obtaining initial approval of a drug, or would support a significant change in the labeling of an approved drug (e.g., new indication, inclusion of comparative safety information); and

(iii) Demonstrate that the clinical trial could not be conducted without charging because the cost of the drug is extraordinary to the sponsor. The cost may be extraordinary due to manufacturing complexity, scarcity of a natural resource, the large quantity of drug needed (e.g., due to the size or duration of the trial), or some combination of these or other extraordinary circumstances (e.g., resources available to a sponsor). (FDA 2009)

(Interestingly, the fourth criterion, present in an earlier draft (that the charge must be “reasonable”), did not make it to the final rule.) Essentially, then, any trial in which patients are to be charged be relevant and well-designed, and must concern a very expensive drug. Some might interpret the “potential clinical benefit” criterion as ruling out most phase 1 P4 trials because they are unlikely to provide any benefit. However, there is at least a chance in some such studies of benefit, and the wording “if demonstrated in the clinical investigations” could also be interpreted as meaning “in this or subsequent trials”.

In addition to these conditions, the FDA acknowledge in the text of their final rule that charging patients for access to investigational agents raises ethical issues:

Two comments stated that there are ethical concerns with charging patients for expanded access use of investigational drugs that may have no benefit and pose safety concerns. Response: In determining whether to permit an expanded access use of an investigational drug, FDA assesses whether the potential risks are reasonable in light of the potential benefits, sometimes on the basis of quite limited clinical evidence. Therefore, FDA agrees that there is a risk that the investigational drug will have no benefit and, therefore, that a patient will pay for an investigational drug that provides no benefit. However, if a drug has a potential benefit that is reasonable in light of the risks associated with the drug, and the sponsor must charge to make the drug available, FDA believes the public health is best served by making the drug available to patients for a fee, even if the potential benefit is not realized in a given patient. FDA believes that the ethical concerns expressed in these comments can be addressed by an informed consent that accurately reflects the costs, potential risks, and potential benefits. (FDA 2009)

However, this again refers to expanded access outside the context of a trial, and the FDA worryingly does not mention that these ethical issues are also raised by (and are perhaps even more important in) payment for participation in a clinical trial.

Emanuel et al. (2015) and Wenner et al. (2015) raise concerns about current regulation of clinical trials being insufficient for governance of P4 trials. In addition to the aforementioned concerns, Emanuel et al. assume that P4 studies would not be peer reviewed: “Peer reviewers can evaluate the potential social benefit of research and prioritize longer-term but more beneficial projects. Pay-to-play funding would prioritize research needs of the wealthy and their ailments…pay-to-play research could also skew researchers and research institutions to pursue lucrative studies that are not necessarily socially valuable.” Similarly, Wenner et al. echo these concerns and contend that the lack of adequate oversight would jeopardise trial quality:

In PFTs, patient sponsors are strongly motivated by the short-term goal of access to new interventions and the profit motive shifts from the sponsor to PFT clinics, which generate revenue directly from the enrollment of participants…This funding model effectively reverses incentives to minimize sample size and encourages sponsors to enroll large cohorts. In doing so, it also increases patient exposure to the risks of unproven interventions…[In non-P4 trials] the threat of regulatory disapproval is leveraged to encourage industry sponsors to utilize “gold standard” methodologies such as blinding and randomization. Peer review plays a similar function for publicly funded studies. Although these mechanisms are imperfect, there is no comparable means to encourage study quality in the PFT model.

Both sets of authors are clearly heavily influenced by their North American perspective. In the United States, trials can be conducted privately without ethics oversight if public funds are not used. In Europe, by contrast, the clinical trial regulation sets out rigorous standards for scientific and ethical review to which all trials must adhere. Any P4 trial conducted by any private or public entity in Europe would be subject to the same rules as any traditional trial. This means that the usual sample size, blinding and randomization standards would be consistently applied (and in any case, it is often the case that more patients than are necessary for a study want to join it). Consequently, almost all of these authors’ regulatory concerns are considerably alleviated in the European context, as illustrated by the recommendations made by Wenner et al.:

First, policy makers could create a mechanism for providing scientific and ethical oversight of PFTs. Second, to encourage the use of such mechanisms in the absence of larger legal or policy mandates, academic medical centers, professional organizations, and licensing boards should discourage their members from participating in studies not approved via such review. Third, policy makers should consider whether accreditation requirements for health care facilities could be used to encourage entities conducting PFTs to utilize an appropriate mechanism for scientific review and ethical.

In the European Union, the mechanism for providing scientific and ethical oversight for P4 trials is already in place in the form of the Clinical Trials Regulation. Adherence to the requirements of the regulation is a legal obligation, so there would be no need to discourage researchers from participating in P4 trials not subject to these standards. Similarly, there would be no need to use accreditation to encourage researchers to use oversight mechanisms required by European law.

Emanuel et al. also suggest that P4 trials raise other new issues for IRBs. They state that when reviewing a P4 trial, “the IRB would have to determine whether the potential direct benefits and knowledge gains from the research justify the financial losses to participants that are associated with enrolling in the trial, as well as whether any risks could be justified if the study might not be able to recruit an adequate number of paying participants and therefore must be abandoned.” However, both of these points are mistaken. First, it is not the role of an IRB to weigh financial loss to participants against the prospect of societal or personal benefit to the patient, even if criteria existed that could make any such weighing valid. Some patients might not be able to afford such a trial, while for others the cost will be relatively negligible. But IRBs do not review the financial circumstances of patients. More importantly, patients in such situations are contributing to funding the research project, not to funding clinical benefit for themselves (though this might not be clear tot hem). Furthermore, if there is a prospect of societal benefit, then even a high amount of money might be “worth it”. Just as IRBs should apply the same rigorous ethical and design standards to P4 trials, they should not let the fact that patients are helping to fund research distract them from traditional risk/benefit analysis. Second, IRBs must always consider for all trials the potential risks of discontinuation due to recruitment failure. These risks might be higher for P4 trials, but an IRB could adjust its considerations accordingly and ensure that patients are adequately informed of any such risk. The only additional information might be to remind patients that their financial contributions are non-refundable.

## Alternative funding mechanisms

Asking patients to pay researchers for participation in trials is an intriguing yet challenging prospect. However, other alternatives may have better prospects of success and ethical acceptability. For example, a variant of the scheme discussed above would be to set it up so that wealthy prospective participants could only be admitted to the trial if they also paid for a worse-off patient to participate. This would mitigate the injustice of asking poorer patients to pay by providing financial assistance to them. However, setting the thresholds between those who can join the trial for free, those who must pay for themselves, and those who must pay for themselves and one (or more?) other would itself pose some challenges.

Another alternative option is “crowdfunding”, via sites similar in function to Kickstarter. Using such schemes, online donors from all over the world could contribute to funding a promising clinical trial in the same way that they can donate online to charities (McNamee 2014). Experiment.com has successfully crowdfunded hundreds of research projects, with many of them in medicine and science. While funding clinical trials in this way is a relatively new idea (Chakradhar 2015), a simple example illustrates how effective it could be. Let’s imagine (using quite low and simple numbers for the sake of simplicity) that we need one million Euros for a clinical trial involving a very expensive drug. If 100,000 people each gave ten Euros, that would generate one million, and possibly in a short space of time. (While such an approach could operate by appealing to altruistic motives, potential contributors could also be given the incentive of a share in the profit from any resulting drug, should the trial be successful.)

This potential solution could fund a trial without directly encountering the aforementioned issues about worsening the therapeutic misconception, coercing patients or adapting designs. This solution offers two main advantages. First, other people who do not have cancer would at least have the opportunity to contribute altruistically. And second, better-off potential participants are likely to contribute more to such a scheme than those who want to donate but are struggling financially (in a parallel to the “pay for yourself plus one” possibility mentioned above). To take a hypothetical example, 20 worse-off potential participants could contribute 1000 Euros each, giving 20,000, 11 very well off participants could each contribute 20,000, giving a total of 220,000, and if 78,000 altruistic donors each gave 10, the target would have been reached. Of course, prospective participants in the trial could themselves take part in any such crowdfunding scheme, though they would not have any guarantee that they would be selected for inclusion in the study.

Although crowdfunding may appear to be a more elegant solution than asking patients to pay, it is not without potential disadvantages. The most obvious is that some crowdfunding efforts simply fail to reach their target, which in this case would mean that the trial could not be funded in this way. Indeed, even if some trials raised enough money via crowdfunding to go ahead, the higher the number of trials seeking funding, the more likely it is that any given one will fail to meet its target. Another concern is that some diseases and types of cancer are easier to fundraise for than others, meaning that it might be impossible to use crowdfunding for less well-known diseases like bowel cancer because all the publicity and funds go to breast and prostate cancer trials. However, if crowdfunding fails, all other avenues would genuinely be exhausted and patients could then be asked to pay themselves, provided that measures were put in place to address the aforementioned ethical concerns.

The most realistic solution is likely to be a combination of crowdfunding and P4. Ideally, crowdfunding will be attempted first, and hopefully raise sufficient funds for a trial. However, if insufficient funds are raised, P4 could be used to raise the remainder of the funds. As well as enabling the trial to go ahead, this option would also enable less well-off people who cannot afford to pay to join the trial, as their places on the trial would be crowdfunded by the raised funds; the same applies to a fully crowdfunded trial. (Using initial crowdfunding to enable socioeconomically disadvantaged patients to participate could even be a regulatory requirement for P4 trials.)

One other possibility would be to “pay it forward to play”. Under any such system, only phase 2, 3 or 4 P4 trials would be permitted, with the funds raised also contributing towards the costs of future phase 1 trials (along with funds raised by a tax on licensed new drugs and some money from the public purse). This would avoid some of the more challenging ethical issues raised by asking patients to pay for participation in phase 1 trials where they are extremely unlikely to get any personal benefit. In essence, only those with a chance of clinical benefit would be able to pay for participation, and their payments would subsidise both their own trial and phase 1 trials for future patients. Ethically, this seems like an attractive option, but it would take considerable investment and development to start up. Furthermore, the aforementioned issues concerning randomization in later-phase trials might also be problematic.

## Conclusion

It is understandable that clinical researchers who want to test an expensive new drug with impressive results from animal studies would be very keen to fund their expensive trials with P4; only a small percentage of potential trials gain funding despite the very high quality of many of those that are rejected. It is also understandable that those who have money and a diagnosis of a disease that has a prognosis that is not good might want to pay for trials of promising drugs. However, doing so further heightens existing concerns about the therapeutic misconception and equity. Emanuel et al. conclude that P4 trials risk “(1) skewing the types of studies pursued as well as the amount of time researchers and facilities devote to particular projects; (2) exploiting potential human subjects; and (3) compromising the methodological rigor of clinical studies.” However, as we have suggested in our analysis, the peer and ethical review structures in place in Europe offer much stronger protections than equivalent mechanisms in the United States, and P4 trials conducted in the European Union would be unable to “slip through the regulatory gap” in the way feared by Emanuel, Wenner and colleagues. Specifically, research ethics committees can prevent skewing of study priorities, the risk of exploitation can be avoided with careful use of existing review and consent mechanisms, and Europe-based researchers seeking to conduct P4 trials are mandated to meet normal trial standards. Therefore, under certain very limited circumstances, a carefully designed trial could perhaps recruit paying participants in an ethical way, but any such trial would have to guard even more than usual against the therapeutic misconception and make it clear to patients that they were paying not for access to a drug, but for participation in research. We do not recommend abandoning usual dose escalation protocols or randomization in order to increase the attractiveness of trials for paying participants, as doing so could both increase risk to patients and weaken the resulting evidence. Our main recommendations are summarized in Table 1. The alternative avenue of crowdfunding clinical trials represents a more ethically straightforward way of funding clinical trials of highly expensive investigational agents, and this option should be explored before asking patients to pay to take part in clinical trials. In most cases a combination of crowdfunding and P4 may be a more realistic option.

**Table 1:** Recommendations for ethical P4 trials

1. Crowdfunding should generally be explored before a P4 trial is considered, and it may be necessary to combine crowdfunding with P4
2. If the worst-off in society tend to benefit in the long run, a temporary inequality to those who cannot afford to participate in a P4 trial may be justified
3. Research ethics committees must ensure that the design of trials is not compromised because of their P4 nature; specifically, all current trial standards regarding blinding, randomization, sample size and dose escalation must be met
4. Extra safeguards against the therapeutic misconception must be put in place: those who “pay to play” are paying for participation in a trial that aims to provide social value through generalisable knowledge. They are paying for involvement in research, and any potential benefit to the patient/participant is merely an unlikely side effect
